# Chemical synthesis of RNA with site-specific methylphosphonate modifications

**DOI:** 10.1016/j.ymeth.2016.03.024

**Published:** 2016-03-30

**Authors:** Sara Flür, Ronald Micura

**Affiliations:** Institute of Organic Chemistry and Center for Molecular Biosciences, CMBI, Leopold-Franzens University, Innrain 80-82, 6020 Innsbruck, Austria

**Keywords:** RNA solid-phase synthesis, Methylphosphonate, Stereochemical assignment, Thermodynamic base pairing stability

## Abstract

Methylphosphonate(mP)-modified RNA serves as valuable probe to evaluate biomolecular interactions between the nucleic acid backbone and binding partners, such as proteins or small molecules. Here, we describe an efficient workflow for the synthesis of RNA with a single mP modification in diastereomerically pure form. While the automated assembly of mP-modified RNA is straightforward, its deprotection under basic conditions is challenging; a carefully optimized step-by-step procedure is provided. In addition, we demonstrate purification and separation strategies for the *R*_P_ and *S*_P_-configurated RNA diastereomers using a combination of anion-exchange and reversed-phase HPLC, and comment on troubleshooting if their separation appears difficult. Furthermore, we demonstrate the stereochemical assignment of short *R*_P_ and *S*_P_ mP-modified RNA diastereomers based on 2D ROESY NMR spectroscopy and we report on the impact of the mP modification on thermal and thermodynamic stabilities of RNA-DNA hybrid and RNA-RNA duplexes.

## 1 Introduction

Ribonucleic acids with methylphosphonate (mP) modifications have attracted significant interest recently to probe ribosome function during EF-G catalyzed elongation by atomic mutagenesis and to study intracellular signaling by second messenger cyclic-di-GMP derivatives that are resistant to phosphodiesterases [1,2]. Thereby, the replacement of a nonbridging phosphate oxygen by a methyl group makes the nucleic acid backbone charge neutral. It furthermore erases the possibility for hydrogen bond formation since proton acceptor capability gets lost [3–8]. Moreover, the mP modification generates chirality at the phosphor atom (*R*_P_ and *S*_P_) [9], and therefore, the encounter of nucleic acids diastereomers is directly associated with the introduction of the methylphosphonate modification.

Historically, Ts’o and coworkers first described the synthesis of dimers of 2′-deoxyribonucleosides with a mP linkage in 1979 [10]. For many years, access to methylphosphonates was limited to dimers or short oligomers of DNA or 2′-OCH_3_ RNA only [3,4,11], although solution and solid-phase syntheses of mP containing nucleic acids were continuously improved by Engels et al. [12], Agarwal et al. [13], Lebedev et al. [14], Heliński et al. [15], Reynolds et al. [4], and Schell et al. [16]. With respect to RNA, it was soon recognized that the 2′-OH in direct neighborhood to a 3′-*O*-methylphosphonate group makes this linkage unstable; strand cleavage is observed as a result of nucleophilic attack of the 2′-OH, providing a 5′-RNA fragment with cyclic 2′, 3′-*O*-methylphosphonate and the corresponding 3′-RNA fragment with a free 5′-OH group. Therefore, stable RNA with mP linkages usually goes along with a 2′-deoxy or 2′-methoxy ribose modification at the site of the methylphosphonate nucleoside [6,17].

For many applications, diastereomerically pure mP-modified nucleic acids are required. Their stereospecific synthesis is achieved by using dimer building blocks that are synthesized involving stereoselective reactions [18]. Alternatively, and more often applied, the racemic mixtures are separated by chromatographic methods [4]. On reversed-phase (RP) high pressure liquid chromatography (HPLC), the elution behavior is such that the *R*_P_-configurated oligonucleotide diastereomer usually elutes prior to the corresponding *S*_P_ diastereomer. The configuration at the phosphor center is frequently assigned by NMR spectroscopy relying on NOESY or ROESY experiments [3,19], but X-ray crystallography is suited as well [1,3,20,21]. Circular dichroism (CD) spectroscopy, however, is only of limited value for the determination of phosphonate configuration [10,19,22,23].

Oligodeoxyribonucleotides (DNA) that contain mP linkages have a neutralized section in their phosphate backbone that can affect stability properties. With respect to thermodynamic base pairing stability, this modification can stabilize but also destabilize duplexes formed with complementary DNA or RNA depending on the site of modification and on the configuration [4,5,19]. By UV-melting temperature experiments, Hamma et al. found that mP linkages in the loop region stabilized a hairpin fold but those in the stem region had the opposite effect [5]. With respect to ‘bio’-stability, oligonucleotides with a mP linkage are highly resistant to nuclease degradation. Furthermore, they efficiently permeate through cell membranes [4,13]. Thus, great potential was seen for their use as regulators of gene expression by the antisense approach where antisense oligonucleotides inhibit translation by binding to mRNA [4,7,24,25]. Other studies included the investigation of B to Z-DNA transition using oligonucleotides containing a mP modification of distinct stereochemistry [11], footprinting of protein-DNA backbone contacts [26], DNA bending upon phosphate neutralisation [27], or an examination of the coordination of cis-plastin to the DNA phosphate backbone [28].

Oligoribonucleotides (RNA) with site-specific mP backbone modifications were for the first time successfully applied in 1993 and 1994 to map the phosphate contacts, that were thought to be critical for RNA recognition by HIV-1 regulatory proteins [6,17]. In 2001, the mP modification was again used to study RNA–protein interactions, namely between the MS2 coat protein and its cognate hairpin [8]. Only very recently, we investigated site-specifically mP-modified RNA to explore the role of a ribosomal RNA phosphate oxygen during the elongation factor G (EF-G)-triggered GTP hydrolysis [1].

The automated solid-phase synthesis and deprotection of DNA and 2′-OCH_3_ RNAs with individual mP backbone groups are straightforward. Synthesis and deprotection can be performed following standard procedures comparable to the unmodified counterparts by using commercially available nucleoside 3′-methylpho sphonamidites that possess either a ribose 2′-deoxy- or 2′-OCH_3_ group [29]. For RNAs with individual mP linkages, the same building blocks have to be used, however, the situation is much more challenging because basic deprotection under standard conditions results in severe degradation of the target mP-RNA and minor yields.

Here, we present reliable procedures for the implementation of a mP linkage into RNA that replaces a natural phosphodiester moiety in an otherwise unaltered backbone. Access to both diastereomers is described. Additionally, the impact of the modification on thermodynamic duplex stability was analyzed and is reported.

## 2 Experimental approach and procedures

**Fig. 1:**
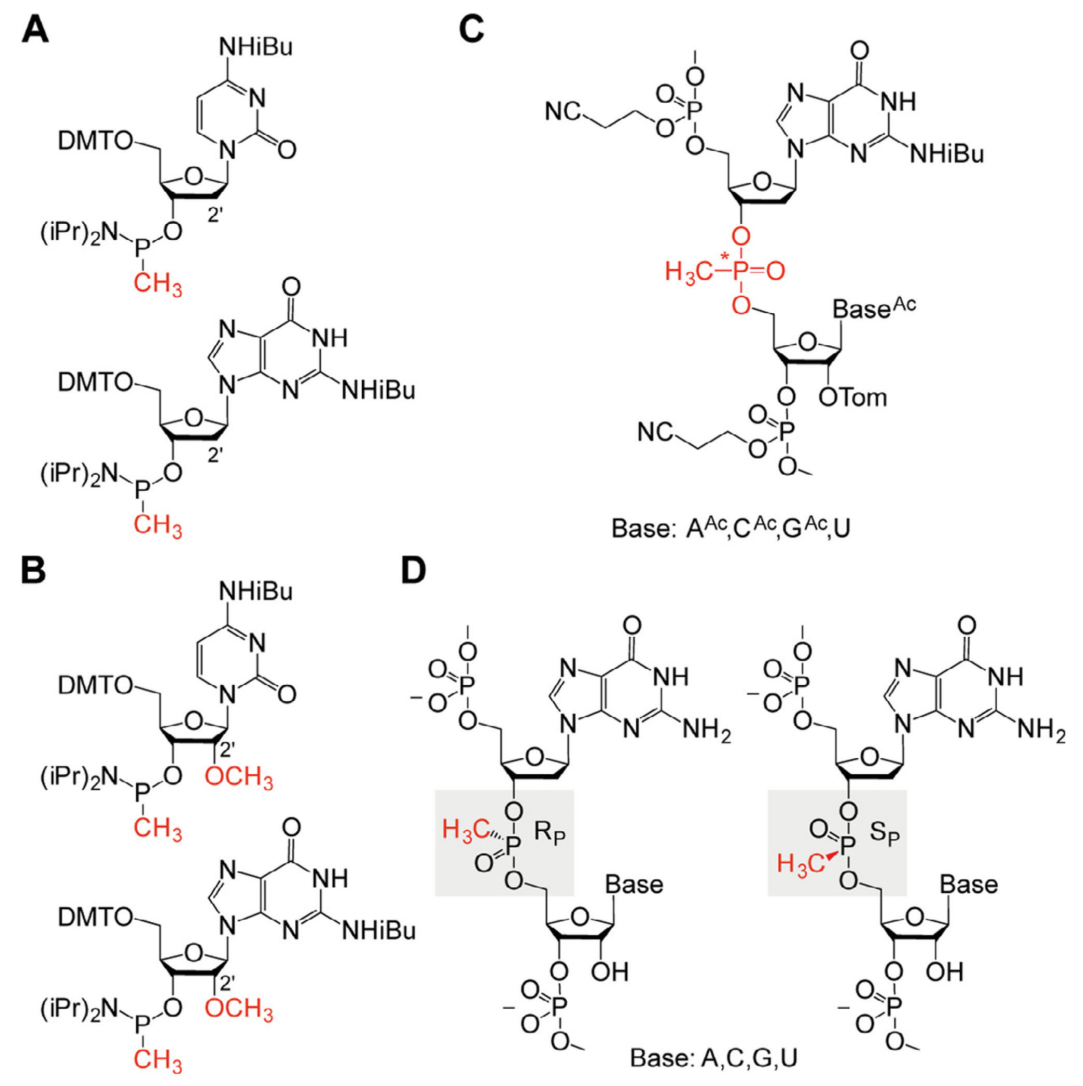
Chemical structures of methylphosphonate backbone modifications for nucleic acids. A) Methylphosphonamidite building blocks for nucleic acid solid-phase synthesis exemplified for 2′-deoxycytidine and 2′-deoxyguanosine; B) Same as (A), but for 2′-OCH_3_ cytidine and 2′-OCH_3_ guanosine; C) Protected RNA (dinucleotide step) with a methylphosphonate modification after assembly of the RNA by automated solid-phase synthesis; (2′-*O*-[(triisopropylsilyl)oxy]-methyl (Tom), asterisk indicates chiral phosphor atom; D) Deprotected RNA (dinucleotide step) with stereochemical assignment of *R*_P_ and *S*_P_ diastereomers.

The preparation of diastereomerically pure mP-modified RNA (Fig. 1) involves three major steps: (*i*) assembly of the protected RNA on solid-phase using nucleoside phosphoramidites in combination with methylphosphonamidite building blocks, (*ii*) deprotection and purification by anion-exchange chromatography to obtain synthetic RNA that contains the mP modification, and finally (*iii*) separation of the *R*_P_ and *S*_P_ mP-RNA diastereomers by reversed-phase chromatography.

### 2.1 Solid-phase synthesis of mP-modified RNA

In modern solid-phase RNA synthesis, protected nucleoside phosphoramidite building blocks are sequentially coupled to a solid support-bound nucleoside. Each cycle starts with a detritylation step (removal of the 5′-*O*-(4,4′-dimethoxy)trityl(DMT) protecting group), followed by a coupling step (activation/coupling) and a capping step, and the last step is the oxidation from P(III) to P(V) (Table 1). Nucleobase acylated and 2′-*O*-[(triisopropylsilyl)oxy]me thyl (Tom) protected standard nucleoside phosphoramidites are commercially available, e.g. from *ChemGenes* or *GlenResearch*, as well as dG and 2′-*O*CH_3_-G methyl phosphonamidites (Fig. 1A, B) and polystyrene support (e.g. from *GE Healthcare*, Custom Primer Support, 80 μmol/g; PS 200). All oligonucleotides are synthesized on a nucleic acid synthesizer (ABI 392) on a 1.5 μmole scale. The solutions of amidites and the solution of the activator 5-(Benzylthio)-1*H*-tetrazole (*Carbosynth*) in acetonitrile are dried over activated molecular sieves (4 Å, *Sigma*-*Aldrich*) overnight. Although 2′-deoxy-5′-*O*-(4,4′-dimethoxytrityl)-*N*^2^-isobutyrylguanosine 3′-[(methyl-(*N,N*-diisopropyl)]-phosphonamidite has to be dissolved in tetrahydrofuran, the corresponding 2′-OCH_3_ guanosine phosphonamidite is applied in acetonitrile. Compared to unmodified phosphoramidites, a longer coupling time for methylphosphonamidites (6 min) is advisable.

### 2.2 Deprotection of mP-modified RNA and cleavage from the solid support

Deprotection and cleavage from the solid support is the most critical step in the preparation of mP-modified RNA (Fig. 1C, D). For cleavage of the base labile groups, previous reports recommend methanolic ammonia [6,17], or alternatively, mixtures of ethanol, acetonitrile, ammoniumhydroxide, and ethylenediamine [8]. Then, removal of the 2′-*O*-silyl groups follows standard conditions, with tetrabutylammonium fluoride (TBAF) in tetrahydrofuran (THF). In our hands, both procedures gave unsatisfying yields and qualities of crude RNA products indicating severe degradation. The detailed protocol below is the result of a comprehensive and careful optimization of deprotection conditions to give high quality crude products.

#### 2.2.1 Materials

Aqueous ammonium hydroxide solution (28% NH_3_ in H_2_O), ethanol, acetonitrile, ethylenediamine.

1 M Triethylammonium bicarbonate (TEAB) stock buffer: Triethylamine (101.2 g, 1 mol) is added to 800 mL nanopure water and CO_2_ is bubbled through until pH 8.0 is obtained. The volume is adjusted to 1000 mL with nanopure water. The buffer solution is stored at 4 °C and diluted to 0.1 M just before use.

1 M Triethylammonium acetate (TEAA) buffer: Glacial acetic acid (60 g, 1 mol, 57 mL) is dissolved in 750 mL of nanopure water. Then, 101 g triethylamine (1 mol, 139 mL) is slowly added and the two layers are mixed carefully in an ice bath. The pH is adjusted to 7.0 with triethylamine. Finally, the volume is adjusted to 1000 mL with nanopure water. The buffer can be stored at 4 °C for several months.

1 M Tetrabutylammonium fluoride trihydrate (Bu_4_NF·3H_2_O) in tetrahydrofuran (THF).

Nanopure water provided by a Millipore-Q System (18 Ω/cm).

Parafilm, 2 mL micro tubes, 50 mL and 100 mL round-bottomed flask, rotary evaporator, high vacuum pump, ÄKTAprime plus system (*GE Healthcare*), size exclusion column (*GE Healthcare*, HiPrep™ 26/10 Desalting; 2.6 × 10 cm; Sephadex G25), HPLC system, Dionex DNAPac^®^ PA-100 column (4 mm × 250 mm).

Stepwise procedure for the deprotection of mP-modified RNA and cleavage form the solid support after synthesis:

(1) Transfer the solid support from the synthesis column into a 2 mL screw cap micro tube.
(2) Add 0.5 mL of a mixture of concentrated aqueous ammonium hydroxide solution (28% NH_3_ in H_2_O*)*/ethanol/acetonitrile (10/45/45, v/v/v) and agitate the mixture for 30 min at room temperature.
(3) Add 0.5 mL of ethylenediamine and agitate the mixture for 5 h at room temperature.
(4) Filter the supernatant into a 100 mL round-bottomed flask and wash the remaining solid support twice with ethanol/water (1/1, v/v). The supernatant and the washings are combined in the 100 mL round-bottomed flask.
(5) Add 1 mL of 1 M triethylammonium bicarbonate (TEAB) buffer (pH 8.0).
(6) The solution is evaporated and a clear oil is obtained. **Note:** The 100 mL flask is recommended due to possible strong foaming of the solution. Rotavapor bath temperature should be set to or below 40 °C. To avoid degradation of the RNA, try to keep evaporation time as brief as possible.
(7) Dry under high vacuum for 20 min.
(8) Add 1 mL of 1 M tetrabutylammonium fluoride trihydrate (Bu_4_NF·3H_2_O) in THF.
(9) The suspension is agitated at 37 °C under light exclusion for 16 h. **Note:** Without agitation of the two phases, the deprotection will not work properly.
(10) Add 1 mL of 1 M of triethylammonium acetate (TEAA) (pH 7.0).
(11) Reduce the volume of the solution by evaporating THF. **Note:** Do not evaporate to dryness.
(12) Apply the remaining solution to a size exclusion column (*GE Healthcare*, HiPrep™ 26/10 Desalting; 2.6 × 10 cm; Sephadex G25) connected to an ÄKTA Prime Plus system (*GE Healthcare*) to get rid of salts. Elute with H_2_O at a flow rate of 1 mL/min and collect the RNA in a 50 mL round-bottomed flask.
(13) The collected fraction containing the RNA is evaporated to dryness using a rotary evaporator. Rotavapor bath temperature should be set to 40 °C or below.
(14) Resuspend the crude RNA in 1 mL of nanopure water (stock solution). **Note:** At this point the sample might be stored at −20 °C for several weeks.

### 2.3 Purification and separation of *R*_P_ and *S*_P_ mP-RNA diastereomers by chromatography

The crude mP-RNA products are analyzed and purified by anion-exchange chromatography. *R*_P_ and *S*_P_ diastereomers are separated by reversed-phase chromatography (Fig. 1D). For both applications, we used a high-pressure-liquid-chromatography (HPLC) system consisting of autosampler (*Dionex* ASI-110), pump (*Dionex* P580), column thermostat (*Jetstream 2 Plus*), and UV detector (*Dionex* UVD-170U).

#### 2.3.1 Anion-exchange HPLC for the analysis of crude mP-RNA products

The crude RNA products are analyzed by anion-exchange chromatography on a *Dionex* DNAPac PA-100 column (4 × 250 mm) at 80 °C. RNA stock solution (2 μL) is diluted to 100 μL and injected into the HPLC system. Flow rate: 1 mL/min; eluent A: 25 mM Tris·HCl (pH 8.0), 6 M urea; eluent B: 25 mM Tris·HCl (pH 8.0), 0.5 M NaClO_4_, 6 M urea; gradient: 0–60% B in A within 45 min or 0–40% B in 30 min for short sequences up to 15 nucleotides; UV detection at 260 nm.

##### 2.3.1.1 Buffer preparation

*Eluent A* (25 mM Tris·HCl (pH 8.0), 6 M urea): Urea (360 g, 6 mol; *Roth*) and 100 ml 250 mM Tris·HCl (pH 8.0) stock solution are dissolved in nanopure water and adjusted to 1000 mL.

*Eluent B* (25 mM Tris·HCl (pH 8.0), 6 M urea, 0.5 M NaClO_4_): Urea (360 g, 6 mol; *Roth*), 100 ml of 250 mM Tris·HCl (pH 8.0) stock solution and sodium perchlorate monohydrate (70.2 g, 0.5 mol) are dissolved in nanopure water and adjusted to 1000 mL.

#### 2.3.2 Anion-exchange HPLC for the purification of *R*_P_/*S*_P_ mP-RNA

The crude deprotected mP-RNA is purified on a semipreparative *Dionex* DNAPac PA-100 column (9 × 250 mm) at 80 °C. Flow rate: 2 mL/min; eluent A: 25 mM Tris·HCl (pH 8.0), 6 M urea; eluent B: 25 mM Tris·HCl (pH 8.0), 0.5 M NaClO_4_, 6 M urea; UV detection: 260 nm. To avoid column overload, small aliquots of the crude 1 mL-RNA stock solution are purified, starting with 20 μL to optimize the gradient (gradient steepness Δ = approximately 8–10% eluent A in B within 20–30 min (for RNA of up to 30 nt)). Fractions containing the pure product are desalted using a C18 Sep-Pak^®^ cartridge. The cartridge is equilibrated with 2 × 10 mL CH_3_CN (HPLC grade), 3 × 10 mL CH_3_CN/H_2_O (1/1, v/v), 3 × 10 mL H_2_O and 3 × 10 mL 0.1 M TEAB (${\left({\text{Et}}_{3}\text{NH}\right)}^{+}{\text{HCO}}_{3}^{-})$ buffer (pH 8.0). The product containing fractions are diluted with an equal volume of 0.1 M TEAB buffer and loaded onto the equilibrated cartridge. After washing 3× with 10 mL H_2_O, the RNA is eluted with 3 × 10 mL CH_3_CN/H_2_O (1/1, v/v) into a 50 mL round bottom flask and evaporated to dryness using a rotary evaporator. The purified RNA is resuspended in 1 mL of nanopure water and the yield is determined by UV photometrical analysis. At this point, the sample can be stored at −20 °C for several months. The integrity of the purified *R*_P_/*S*_P_ RNA is analyzed by liquid chromatography–electro spray ionization-mass spectrometry (LC–ESI-MS).

#### 2.3.3 Reversed-phase HPLC for the separation of *R*_P_ and *S*_P_ diastereomers of mP-RNA

Analysis of *R_P_* and *S_P_* mP-RNA diastereomers is performed by reversed-phase chromatography on a Waters XBridge C18 column (130 Å, 5.0 μm, 4.6 × 150 mm) at 40 °C. An aliquot of *R_P_*/*S_P_* RNA (about 200 pmol; ~2 μL) from the 1 mL-stock solution is diluted into 100 μL nanopure H_2_O and injected. Flow rate: 1 mL/min; eluent A: 0.1 M ammonium acetate, pH 7.0; eluent B: acetonitrile (HPLC grade); gradient: steepness Δ 3–5% B in A within 25 min; UV detection at 260 nm.

##### 2.3.3.1 Buffer preparation

*Eluent A* (0.1 M Ammonium acetate, pH 7.0): A 1 M stock solution is prepared. Ammonium acetate (77.1 g; 1 mol) is dissolved in 800 mL nanopure water and the pH is adjusted to 7.0 with an ammonium hydroxide solution (28% NH_3_ in H_2_O). The volume is adjusted to 1000 mL with nanopure water and the stock solution was stored at 4 °C. Finally, 0.1 M ammonium acetate buffer is prepared by diluting 100 mL of the stock solution with 900 mL of nanopure water. The buffer can be stored at 4 °C for several months.

*Eluent B:* Acetonitrile (HPLC grade, *VWR*).

The mP-RNA diastereomers are separated on a reversed-phase *GE Healthcare* Resource RPC (3 mL; 6.4 × 100 mm) column because of higher loading capacity, at 40 °C. Flow rate of 2 mL/min; eluent A: 0.1 M ammonium acetate, pH 7.0; eluent B: acetonitrile (HPLC grade); UV detection: 260 nm. To optimize the gradient for separation, only about 20 μL of the stock solution is used. In general, gradients in the range of 0–4% B in A within 25 min for sequences of up to 30 nucleotides are well suited. About 100 μL-aliquots of the stock solution can be injected without overloading of the column. Fractions containing the diastereomerically pure RNA are collected in falcon tubes, diluted with an equal volume of 0.1 M TEAB buffer (pH 8.0) and loaded onto a C18 Sep-Pak^®^ cartridge (equilibrated by washing with 2 × 10 mL CH_3_CN, 3 × 10 mL CH_3_CN/H_2_O (1/1, v/v), 3 × 10 mL H_2_O and 3 × 10 mL 0.1 M TEAB buffer). After rinsing the loaded column three times with 10 mL H_2_O, the diastereomerically pure RNA was eluted with 30 mL CH_3_CN/H_2_O (1/1, v/v) into a 50 mL round bottom flask and evaporated to dryness.

The *R*_P_ and *S*_P_ mP-RNAs, respectively, are each dissolved in 1 mL of nanopure H_2_O; these stock solutions can be stored at −20 °C for months. Yields are determined by UV photometrical analysis. To check the diastereomerical purity, 200 pmol of each separated diastereomer is re-analyzed on the XBridge™ C18 column (130 Å, 5.0 μm, 4.6 × 150 mm) at 40 °C. Flow rate: 1 mL/min; eluent A: 0.1 M ammonium acetate, pH 7.0; eluent B: acetonitrile (HPLC grade); gradient: steepness 3–5% B in A within 25 min; UV detection at 260 nm.

### 2.4 Mass spectrometry of mP-modified RNA

The molecular weights of the purified oligonucleotides are analyzed by mass spectrometry (Table 2), using an ion trap instrumentation (Finnigan LCQ Advantage MAX; Thermo Fischer Scientific) connected to a micro LC system (Amersham Ettan, GE healthcare). The RNA sample is measured in the negative-ion mode with a potential of −4 kV applied to the spray needle. To obtain highquality mass spectra, ethylenediaminetetraacetic acid was added as a chelating agent to remove divalent metal cations which otherwise can form stable adducts with the RNA. LC: sample (200 pmol RNA dissolved in 30 μL of 20 mM EDTA solution; average injection volume: 30 μL); column (Waters XTerra^®^MS, C18 2.5 μm; 1.0 × 50 mm) at 21 °C; flow rate: 30 μL/min; eluent A: 8.6 mM Et_3_N, 100 mM 1,1,1,3,3,3-hexafluoroisopropanol in H_2_O (pH 8.0); eluent B: methanol; gradient: 0–100% B in A within 30 min; UV detection at 254 nm.

### 2.5 NMR spectroscopy for stereochemical assignments

The RNA sample is lyophilized and dissolved in D_2_O containing 10 mM KH_2_PO_4_ and 50 mM KCl, pH 6.4. The final RNA concentration is about 0.2 mM. The ^1^H 1D-NMR spectra are acquired using a double pulsed field gradient spin–echo pulse sequence. The ROESY ^1^H,^1^H NMR spectra are acquired on a 600 MHz spectrometer (Bruker Avance II+) equipped with a proton-optimized triple resonance NMR ‘inverse’ (TCI) CryoProbe Prodigy (5 mm). The ROESY spectra are acquired at 278 K. The size of the data matrices are 2048 × 300 complex data points, the number of scans is 144, the interscan delay is 1.5 s, and the mixing time is set to 150 ms, resulting in a total measuring time of 20 h for each spectrum.

### 2.6 UV melting experiments for analysis of thermodynamic stabilities

An aliquot of the RNA sample is taken from the stock solution and lyophilized to dryness. Melting buffer (10 mM Na_2_HPO_4_, pH 7.0, 150 mM NaCl) is added to give the desired concentration and the solution is transferred into a quartz cuvette (1 cm [800 μL] or 0.1 cm [300 μL] path (Hellma)). The samples are degassed for 2 min in an ultrasound bath and then covered with a layer of silicon oil (dimethylpolysiloxane) to avoid evaporation during the measurements at elevated temperatures. Absorbance versus temperature profiles are recorded at 250 and 260 nm on a UV–VIS spectrophotometer (Varian Cary 100) equipped with a multiple cell holder and a peltier temperature control device. Data are collected after a complete cooling and heating cycle at a rate of 0.7 °C/min. In general, melting curves are measured at five different concentrations ranging from about 1 to 40 μM for bimolecular melting transitions and at two different concentrations for monomolecular melting transitions. KaleidaGraph (Synergy software) was used for the analysis of UV melting profiles. The accuracy of the melting point determination by this method is ±0.3–0.5 °C.

## 3 Results and discussion

**Fig. 2:**
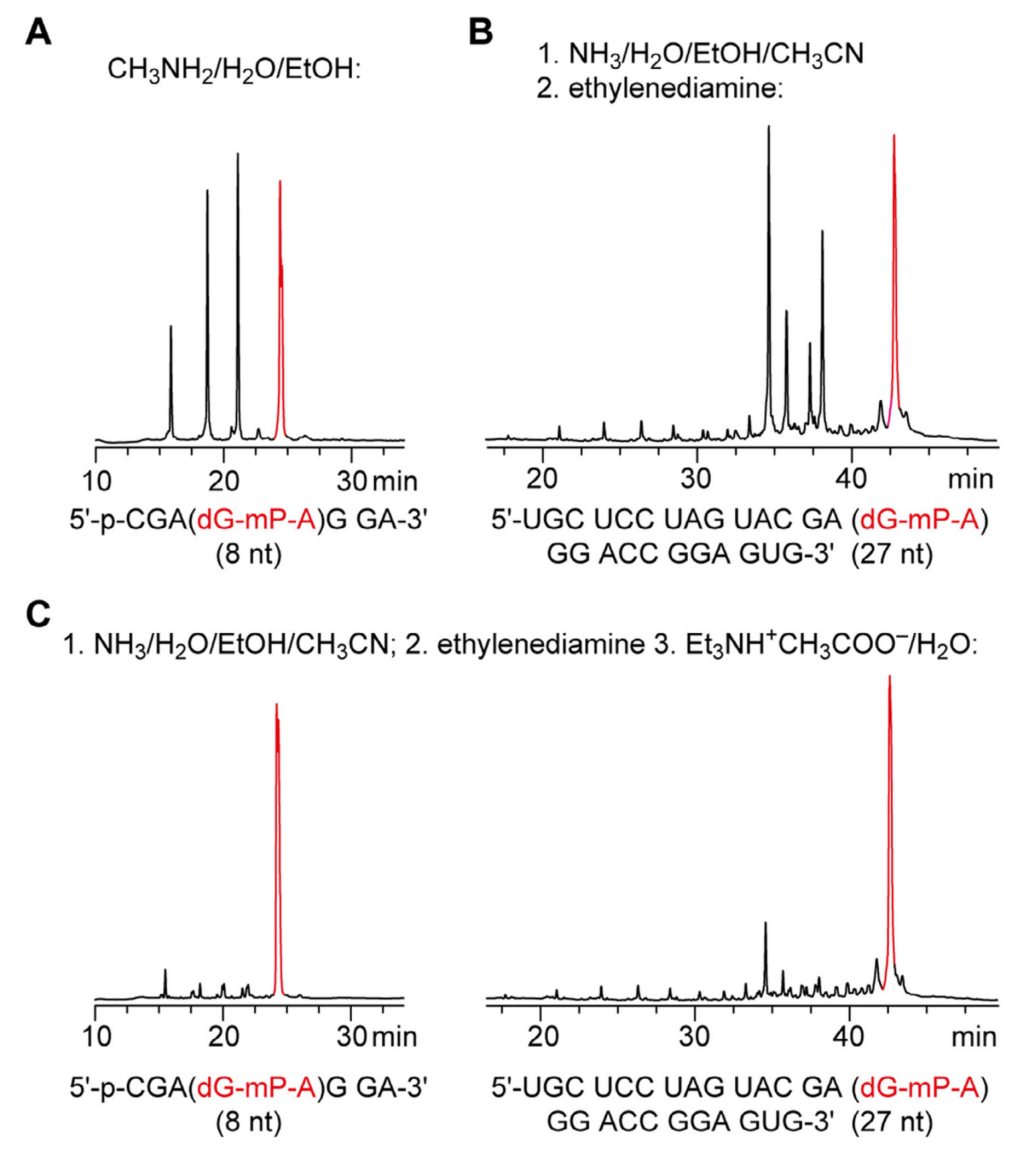
Comparison of different deprotection procedures of mP-modified RNA analyzed by anion exchange HPLC. A) Crude deprotected material after deprotection using standard RNA deprotection protocols (for conditions see main text); B) Crude deprotected material after deprotection using a deprotection protocol reported in the literature (for details see Ref. [8]); C) Crude deprotected material after deprotection using the optimized protocol reported here (for detailed conditions see main text).

The preparation of diastereomerically pure RNA with a single mP backbone modification involves several steps. While the automated solid-phase synthesis of these RNA derivatives follows standard synthesis cycles that do not require adaptations and utilize commercially available 2′-*O*-TOM protected nucleoside phosphoramidites in combination with commercially available methylphosphonamidite building blocks, the subsequent deprotection and cleavage from the solid support can be considered most critical. Unmodified RNA is routinely deprotected by first applying methylamine in ethanol/water (EMAM), or alternatively, methylamine and ammonia in water (AMA) [30]. Under these conditions, the base labile protecting groups are removed from the RNA very fast and cleanly; importantly, acetyl (instead of benzoyl) protection has to be used for the exocyclic *N*^4^ amino group to avoid transamination at C4 by methylamine [31,32]. Secondly, the 2′-*O*-TOM groups are deprotected by tetrabutylammonium fluoride in tetrahydrofuran [30]. However, when these standard conditions were applied to a short dGmP-containing RNA, we observed major degradation products (Fig. 2A), even when exposure of the synthesized RNA to the basic conditions was reduced to one hour at 35 °C. We therefore set out to find more powerful conditions. The use of concentrated ammonium hydroxide appeared not to be an option because the deprotection of mP-modified DNA was described previously to be accompanied by severe degradation [29]. Also, our attempts to apply aqueous 25% hydrazine solutions (as previously used for the deprotection of pyranosyl-RNA) [33] gave crude products of unsatisfying quality. We therefore became inspired by a protocol by Dertinger et al. who pre-treated the synthesized RNA with a mixture of ethanol, acetonitrile, and ammonium hydroxide solution, and then applied ethylenediamine for several hours, followed by desalting using NAP25 size exclusion columns [8]. This procedure gave much better results with less degradation products (Fig. 2B). Importantly, we were able to significantly improve the procedure by simply diluting the reaction mixture with triethylammonium acetate buffer (1 M) before rapid concentration on a rotary evaporator prior removal of the RNA silyl protecting groups (Fig. 2C). Noteworthy, applying standard conditions with tetrabutylammonium fluoride in tetrahydrofuran (as for unmodified RNA) properly removed all 2′-*O*-TOM groups of mP-modified RNA and did not require further optimization [30].

To further demonstrate the effectiveness of the optimized procedures, they were tested and confirmed for a 27 nt RNA of the same sequence but containing a 2′-OCH_3_G–mP–A instead dG–mP–A linkage (data not shown). We also note that triethylammonium bicarbonate buffer can be replaced by triethylammonium acetate buffer leading to the same results.

**Fig. 3:**
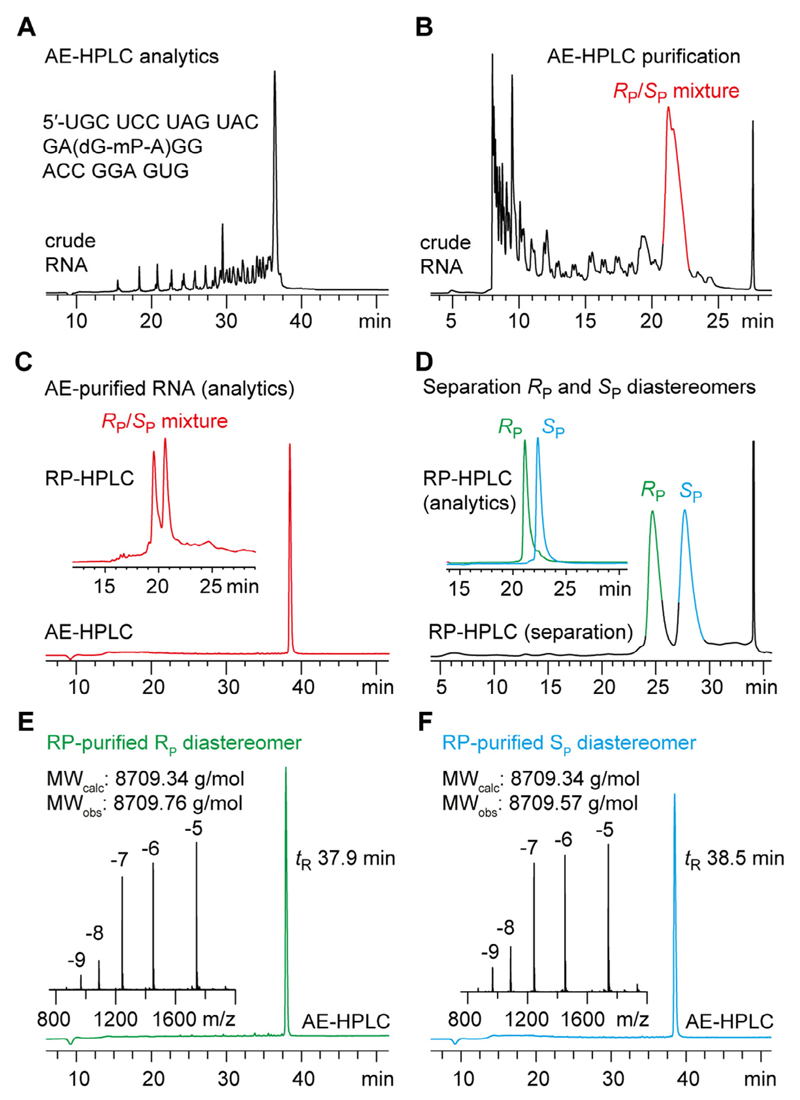
Workflow for the preparation of mP-modified RNA, exemplified for UGC UCC UAG UAC GA(dG-mP-A)GG ACC GGA GUG (27 nt). A) Analytical AE-HPLC trace of crude deprotected RNA; B) Semipreparative AE-HPLC trace of deprotected mP-RNA during purification; product-containing fraction is indicated in red; C) Analysis of the purified RNA by AE- and RP-HPLC reveals that diastereomers can be separated by RP chromatography. D) RP-HPLC separation of *R*_P_ and *S*_P_ diastereomers and analytics of the individual mP-RNA diastereomers (inset). E) AE-HPLC analysis and LC–ESI-MS data of the purified *R*_P_ diastereomer. F) Same as (E), but for *S*_P_ diastereomer (for detailed conditions see main text).

Purification of chemically synthesized RNA is efficiently performed by strong anion-exchange chromatography (AE-HPLC) under denaturating conditions (pH 8.0, 6 M urea) at elevated temperatures (60–80 °C). In our experience, these conditions are especially powerful to separate slightly shorter (n-1, n-2, …) strands from the target RNA, in particular for RNAs larger than 15–20 nucleotides. The same conditions are also successfully applied to analyze (Fig. 3A) and purify large amounts of crude deprotected mP-modified RNA (Fig. 3B). One drawback of AE-HPLC, however, is that the *R*_P_ and *S*_P_ diastereomers of mP-modified RNA are migrating with almost identical retention times, in particular, when the RNAs are longer than 5–10 nucleotides (Fig. 3C).

We therefore recommend to utilize reversed-phase chromatography (RP-HPLC) for the separation of the AE-purified *R*_P_/*S*_P_ mixture of mP-modified RNA. Two buffer systems, namely triethylammonium acetate (TEAA)/acetonitrile (ACN) buffer (pH 7.0) and ammonium acetate (NH_4_OAc)/ACN buffer (pH 7.0) were evaluated applying different gradients and temperatures. Interestingly, we found that the NH_4_OAc/ACN buffer system had better separation power in particular for RNA with more than 15–20 nucleotides (Supporting Fig. S1).

While for short mP-modified RNAs (<10 nt), the use of a rather flat gradient for the NH_4_OAc/ACN buffer system (0–4% B in A within 25 min) also allowed for the direct separation of the crude deprotected RNA products by RP-HPLC (Supporting Fig. S2) with no need to first isolate the mixture of *R*_P_/*S*_P_ diastereomers by AE-HPLC, this usually does not work out for larger mP-modified RNAs as illustrated in Fig. 3.

Furthermore, we note that for the separation of 2′-OCH_3_ mP-modified RNA HPLC-conditions comparable to the 2′-deoxy mP-modified counterparts can be applied. However, in general, we observed less difference in retention time between *R*_P_ and *S*_P_ 2′-OCH_3_ mP-RNA diastereomers and therefore their separation required slightly shallower gradients to achieve baseline-separation.

To summarize, the NH_4_OAc/ACN buffer system on a reversed-phase GE Healthcare Resource RPC (3 mL; 6.4 × 100 mm) column at 40 °C with a flow rate of 2 mL/min using different gradients has proven very powerful for separation of mP-modified oligoribonucleotides of a length of up to 30 nucleotides. However, as for phosphorothioates [34,35], the likelihood for successful separation of diastereomers is sequence dependent, and seemingly inseparable mixture of mP-RNA diastereomers may require extensive tests of different HPLC conditions with alternative buffer system on diverse reversed-phase columns. Moreover, we found for mP-modified (as for thiophosphate-modified) RNA, that the *R*_P_ mP-RNA diastereomer generally elutes before the *S*_P_ diastereomer on reversed-phase C18 columns [5,8,35–38].

**Fig. 4:**
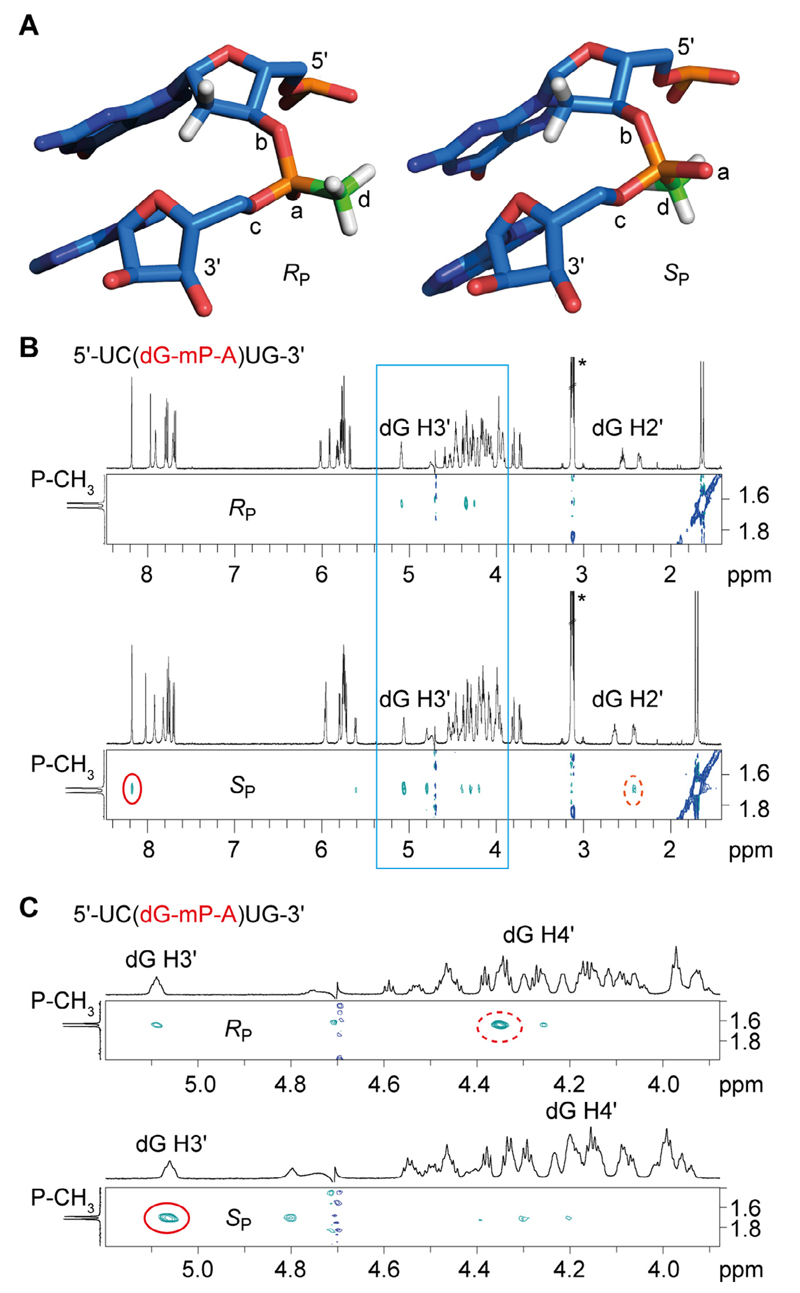
NMR spectroscopic analysis of UC(dG-mP-A)UG. A) 3D model of *R*_P_ and *S*_P_ diastereomers. B) Diastereomeric assignment was based on ^1^H,^1^H ROESY spectroscopy showing crosspeaks of the *S*_P_ methyl group and aromatic nucleobase protons (red circle) as well as one of the 2′-deoxyribose hydrogen atoms (red dotted circle). These cross peaks are lacking for the *R*_P_ diastereomer. C) Expansion of the ^1^H,^1^H ROESY spectrum (corresponding to the blue frame in (B)). The *S*_P_ methyl group gives a more pronounced crosspeak to the 3′-deoxyribose hydrogen atom while the *R*_P_ methyl group shows a more pronounced crosspeak to the 4′-deoxyribose hydrogen atom. Conditions: c(RNA) = 0.2 mM; D_2_O, 10 mM KH_2_PO_4_ and 50 mM KCl, pH 6.4.

The stereochemical assignment of *R*_P_ and *S*_P_ mP-modified RNA diastereomers is reliably performed by NMR spectroscopy as originally described by Löschner et al. for mP-modified 2′-deoxydinucleosides [3]. Here, we exemplified the stereochemical assignment for the short single-stranded RNA 5′-UC(dG-mP-A)UG by nuclear Overhauser enhancement (NOE) derived 2D NMR ROESY ^1^H,^1^H NMR measurements (Fig. 4). The two diastereomers depict distinct signal patterns. While the *S*_P_ diastereomer shows crosspeaks from the methylphosphonate methyl group to aromatic protons of the neighboring nucleobases and to protons at the 2′-position of deoxyguanosine (dG H2′), the *R*_P_ diastereomer does not show any of these correlations (Fig. 4B). Furthermore, the NOE pattern between the methylphosphonate methyl group and the dG H3′ and dG H4′ is complementary for the two diastereomers, with the *S*_P_ diastereomer reflecting a more pronounced correlation to dG H3′ and the *R*_P_ diastereomer showing a more pronounced correlation to dG H4′ instead (Fig. 4C). These observations are consistent with distances measured between 2′-deoxyribose protons and the methylphosphonate methyl group of a non-optimized stacked dinucleotide model (Fig. 4A). For the *R*_P_ diastereomer, the P-methyl group is about 5 Å distant to dG H2′, 4 Å to dG H3′, and 5 Å to dG H4′. For the *S*_P_ diastereomer, the P-methyl group is about 4 Å distant to dG H2′, 2 Å to dG H3′, 5 Å to dG H4′ and 4 Å to H-C8 of guanine.

As second example, the 8nt RNA 5′-p-CGA(dG-mP-A)GGGA was investigated and a very similar ROESY correlation pattern obtained that allowed for unambiguous stereochemical assignment (Supporting Fig. S3). We also note, that we assigned the *R*_P_ and *S*_P_ diastereomers of a 27 nt sarcin-ricin RNA motif of 27 nucleotides on the basis of NOESY ^1^H,^1^H NMR data [1]. There, the mP-modification resided in the loop region. The correlation pattern was again comparable to the smaller RNAs described above. In addition, the assignments were unequivocally confirmed by an independent X-ray structure analysis of the *R*_P_-diastereomer of the same RNA [1].

**Fig. 5:**
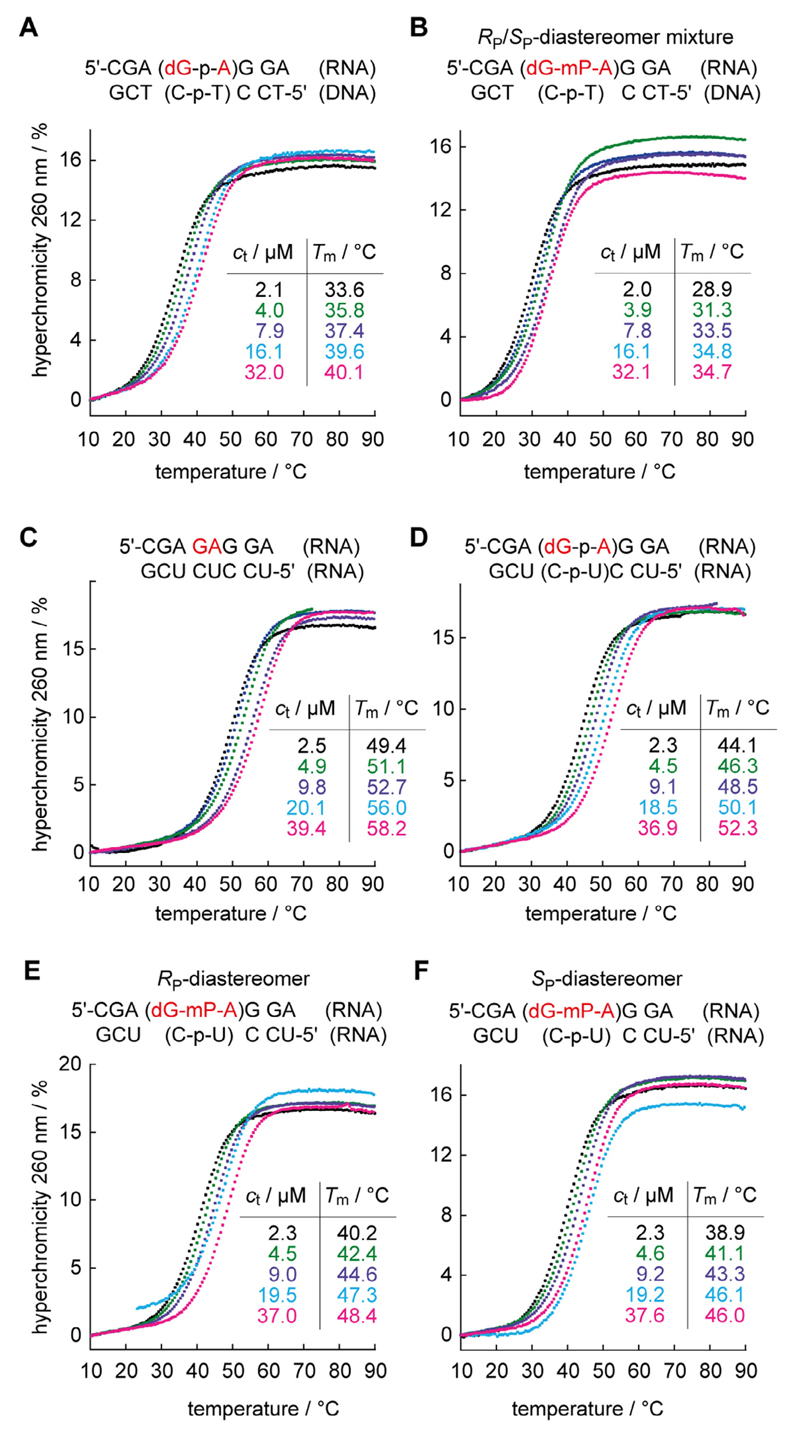
UV melting profiles and thermal stabilities of nonmodified and methylphosphonate containing oligonucleotide duplexes of the same base sequence; for detailed discussion see the main text. Conditions: 10 mM Na_2_HPO_4_, 150 mM NaCl (pH 7.0).

Methylphosphonate backbone modifications impact on the base pairing stability of nucleic acids. Studies on the thermodynamic stabilities of 2′-deoxy- or 2′-methoxy mP-containing oligonucleotides are rare in literature [4,5,18–20,23,39], and often cover very selected aspects for a specific sequence context only. Therefore, we aimed at a more systematic comparative approach as depicted in Fig. 5, based on the analysis of UV melting profiles. We focused on non-selfcomplementary duplexes to determine the impact of a single mP modification at the central GA step of the hybrid duplex 5′-r(CGA*GA*GGA)/5′-d(TCCTCTCG) (Fig. 5A, B) and of the RNA duplex 5′-r(CGA*GA*GGA)/5′-r(UCCUCUCG) (Fig. 5C, D). The thermal (*T*_m_) and thermodynamic data (ΔH, ΔS, ΔG) in aqueous buffer system (10 mM Na_2_HPO_4_, 150 mM NaCl, pH 7.0) were derived from the slope and intercept of the linear regression of the data points in a 1/T_M_ versus (ln c_total_) plot; these data are summarized in Table 3. The RNA-DNA hybrid duplex with the central dG-p-A step melts at 35.6 °C while the corresponding counterpart with a dG-mP-A step melts at 31.2 °C, corresponding to a 4.4 °C decrease in thermal stability. The unmodified RNA-RNA duplex reveals a *T*_m_ value of 50.6 °C which is decreased by 4.8 °C when the central G-p-A sequence is replaced by dG-p-A (*T*_m_ = 45.8 °C). When the charged phosphodiester is replaced by the neutral methylphosphonate group, stability is further diminished. The reduction depends on the mP configuration. The *R*_P_ diastereomer forms a more stable duplex (*T*_m_ = 42.0 °C) compared to the *S*_P_ diastereomer (*T*_m_ = 40.7 °C), with a difference in *T*_m_ of 1.3 °C. Noteworthy, when we measured the duplex stability using the 1:1 mixture of diastereomers, the *T*_m_ value was found to be approximately the arithmetic mean of the *T*_m_ values measured for the two separated isomers (*T*_m_ = 41.4 °C) (data not shown).

At this point we mention that we previously investigated the thermal stabilities of the sarcin-ricin (SRL) stem loop RNA with a mP modification in the loop region [1]. While the unmodified SRL RNA melted around 58 °C, the *R*_P_ and *S*_P_ diastereomer melted slightly higher at 59 °C and 60 °C, respectively. However, no thermodynamic data was extracted because of the non-two-state melting of this system.

Taken together, our data confirms that the mP modification is destabilizing when positioned in a double helical base-paired region while it has a minor effect when positioned in a single stranded loop region. Moreover, the *R*_P_-configurated methylphosphonate retains higher stability compared to the *S*_P_-configurated methylphosphonate.

## 4 Conclusion

In this article, we have described an efficient workflow for the synthesis of RNA with a single mP backbone modification in diastereomerically pure form. Synthesis of mP-modified RNA is straightforward. We have elaborated powerful conditions for the most critical step that is basic deprotection of the synthesized RNA under concomitant cleavage from the solid-support; a detailed step-by-step procedure is provided. In addition, we have demonstrated a path to purify and separate the *R*_P_ and *S*_P_-configurated diastereomers by anion exchange and reversed-phase HPLC and provide troubleshooting comments if their separation appears difficult. Furthermore, we have outlined the stereochemical assignment for short *R*_P_ and *S*_P_ mP-modified RNA diastereomers by 2D ROESY NMR and have reported on the influence that the mP modification exerts on thermal and thermodynamic stabilities of DNA-RNA hybrid and RNA-RNA duplexes.

## Appendix A. Supplementary data

Supplementary data associated with this article can be found, in the online version, at http://dx.doi.org/10.1016/j.ymeth.2016.03.024.

Supporting Fig. S1

Supporting Fig. S2

Supporting Fig. S3

## Figures and Tables

**Table 1:** Conditions for a complete coupling cycle using 2′-*O*-TOM RNA chemistry on 1.5 μmol scale.

Step	Conditions
Detritylation	4% Dichloroacetic acid in 1,2-dichloroethane, 80 s
Activation/Coupling	Phosphoramidites/acetonitrile (0.1 M × 130 μL) and benzylthiotetrazole/acetonitrile (0.3 M × 360 μL), 2 min for standard amidites and 6 min for methylphosphonamidites
Capping	Cap A/Cap B = 1/1 with Cap A: 4-(*N*,*N*′-dimethylamino) pyridine in acetonitrile (0.5 M) and Cap B: Ac_2_O/symcollidine/acetonitrile (2/3/5) (3 × 0.4 min)
Oxidation	I_2_ (20 mM) in THF/pyridine/H_2_O (35/10/5) (1 min)

**Table 2:** Selection of RNAs synthesized with a single methylphosphonate moiety.

RNA Sequence (5′-to-3′direction)	nt	m.w._calc_	m.w_obs_
p-CGA(dG-mP-A)GGA	8	2673.68	2673.62
p-CGA(2′-OCH_3_-G-mP-A)GGA	8	2703.72	2703.51
UGC UCC UAG UAC GA(dG-mP-A)GG ACC GGA GUG	27	8709.34	8709.76
UGC UCC UAG UAC GA(2′-OCH_3_G-mP-A)GG ACC GGA GUG	27	8739.38	8739.86
UC(dG-mP-A)UG	6	1857.20	1857.05
AC(dC-mP-Puromycin)	4	1393.09	1392.79
AC(2′-OCH_3_-C-mP-Puromycin)	4	1423.12	1422.84

**Table 3:** Comparative thermal and thermodynamic data of short oligonucleotide duplexes to evaluate the impact of a single methylphosphonate moiety.

Duplex sequence	*T*_m_ (4 μM) [°C]	ΔH [kcal mol^−1^]	ΔS [cal mol^−1^ K^−1^]	ΔG [kcal mol^−1^]
5′-CGA(dG-p-A)GGA/5′-d(TCCTCTCTG)	35.6	−66.2	−187	−10.5
5′-CGA(dG-mP-A)GGA/5′-d(TCCTCTCTG)	31.2	−66.0	−190	−9.5
5′-CGAGAGGA/5′-UCCUCUCUG	50.6	−65.3	−174	−13.4
5′-CGAdGAGGA/5′-UCCUCUCUG	45.8	−70.5	−193	−12.8
5′-CGA(dG-p-A)GGA (*R*_P_)/5′-UCCUCUCUG	42.0	−65.8	−181	−11.8
5′-CGA(dG-mP-A)GGA (*S*_P_)/5′-UCCUCUCUG	40.7	−58.7	−160	−11.1
