# Supplementary figures and images for: Chemical synthesis of RNA with site-specific methylphosphonate modifications
Source: Methods. Author manuscript; Available in PMC 2017 Apr 26. (PMC5405801; doi:10.1016/j.ymeth.2016.03.024)

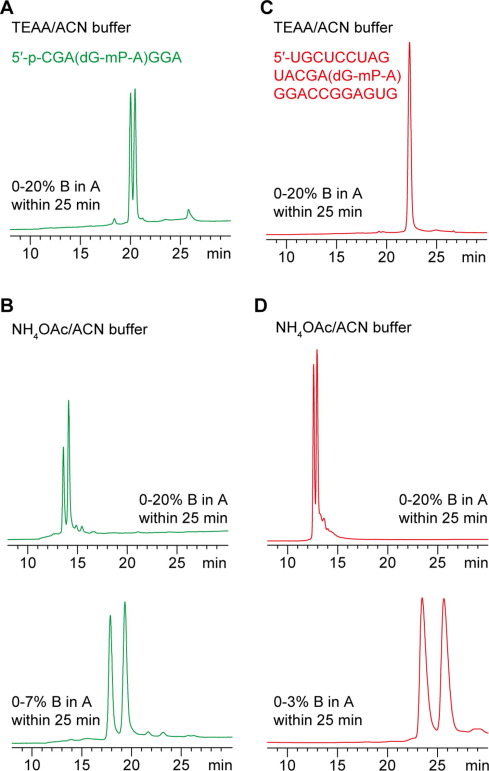

Supplement: Supporting Fig. S1 [file NIHMS72445-supplement-Supporting_Fig__S1.jpg]

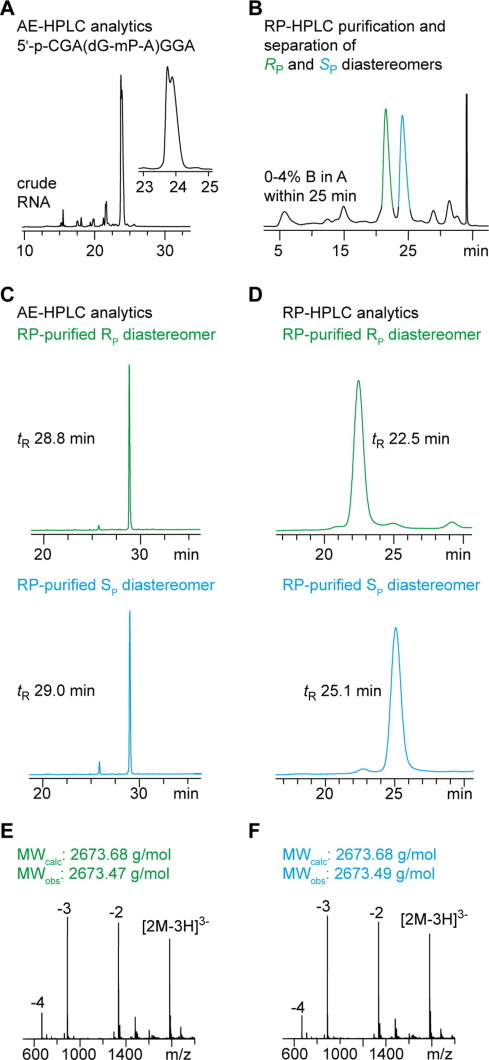

Supplement: Supporting Fig. S2 [file NIHMS72445-supplement-Supporting_Fig__S2.jpg]

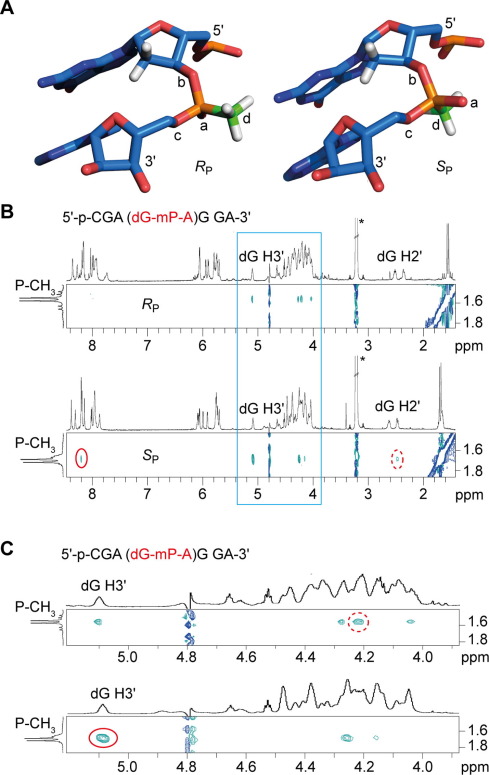

Supplement: Supporting Fig. S3 [file NIHMS72445-supplement-Supporting_Fig__S3.jpg]
